# Is Ivermectin Effective in Treating COVID-19?

**DOI:** 10.3389/fphar.2022.858693

**Published:** 2022-06-21

**Authors:** Shuangshuang Yang, Shan Shen, Ning Hou

**Affiliations:** ^1^ Department of Pharmacy, Shandong Provincial Hospital Affiliated to Shandong First Medical University, Jinan, China; ^2^ Graduate Department, Shandong First Medical University (Shandong Academy of Medical Sciences), Jinan, China

**Keywords:** ivermectin, COVID-19, SARS-CoV-2, antiviral, therapeutic agent

## Abstract

Coronavirus disease 2019 was first discovered in December 2019 and subsequently became a global pandemic with serious political, economic, and social implications worldwide. We urgently need to find drugs that can be effective against COVID-19. Among the many observational studies, ivermectin has attracted the attention of many countries. Ivermectin is a broad-spectrum antiparasitic drug that also has some antiviral effects. We reviewed studies related to ivermectin for the treatment of COVID-19 over the last 2 years (2019.12–2022.03) *via* search engines such as PubMed, Web of Science, and EBSCOhost. Seven studies showed a lower mortality rate in the ivermectin group than in the control group, six studies found that the ivermectin group had a significantly fewer length of hospitalization than the control group, and eight studies showed better negative RT-PCR responses in the IVM group than in the control group. Our systematic review indicated that ivermectin may be effective for mildly to moderately ill patients. There is no clear evidence or guidelines to recommend ivermectin as a therapeutic agent for COVID-19, so physicians should use it with caution in the absence of better alternatives in the clinical setting, and self-medication is not recommended for patients.

## 1 Introduction

Coronavirus disease 2019 (COVID-19) is caused by severe acute respiratory syndrome coronavirus 2 (SARS-CoV-2), and its clinical manifestations are mainly classified as asymptomatic, with mild flu-like symptoms, severe, and fatal. As of 3 April 2022, just over 489 million cases and over 6 million deaths have been reported globally (World Health Organization, 2022). This pandemic has dealt a very serious blow to economic, social, and global sanitary. China has taken strict quarantine and blockade measures, large-scale nucleic acid testing, and community-wide vaccination to combat the pandemic and has adhered to the goal of “dynamic zero”. Among the new coronavirus variants, the Alpha variant (B.1.1.7 spectrum), the Delta variant (B.1.617.2 spectrum), and the Omicron variant (B.1.1.529 spectrum) are the most representative, and they are all classified by the World Health Organization (WHO) as high concern variants. Although Omicron may exhibit milder symptoms after infecting humans, with a 59% and 69% lower risk of hospitalization and death, respectively, than Delta, they are more transmissible and have a partial immune escape (Nyberg et al., 2022). Since 2022, Omicron has continued to spread globally, gradually replacing the previously globally prevalent delta strain.

Ivermectin (IVM) is a broad-spectrum antiparasitic agent approved by the FDA, which demonstrated antiviral activity against several DNA and RNA viruses (Formiga et al., 2021). The antiviral mechanism of action of IVM is to inhibit nuclear import of host and viral proteins, thereby inhibiting viral replication; the most common dose of its tablet administration in clinics is 200 μg/kg. IVM is widely distributed throughout the body after absorption and has the highest concentration in the liver and adipose tissue and is usually oxidized to metabolites in the liver. The most common adverse reactions include elevated transaminases, nausea, diarrhea, dizziness, decreased leukocyte count, allergic reactions, and ocular impairment (Ozer et al., 2022). In the clinical reports, it has been found that the use of IVM will also produce self-limiting ototoxicity, particularly manifested as vestibular lesions (Little and Cosetti, 2021). There are many discussions on its effect in controlling the course and restoring health in patients with COVID-19. Caly et al. (2020) in Australia tested a monkey kidney cell CDw150 *in vitro* and found that 5 μM single-dose IVM could inhibit the RNA replication of the SARS-CoV-2 virus *in vitro* within 24–48 h and reduce it by 5,000-fold (Heidary and Gharebaghi, 2020). This study has attracted extensive attention all over the world. Importantly, the drug concentration used in the study (5 µM) to block SARS-CoV-2 was 35-fold higher than the one approved by the FDA for the treatment of parasitic diseases, which cannot be achieved in a real-world clinic. Therefore, we conducted a systematic review to provide a basis and reference for the promising drug for further clinical decisions and medication precisely.

## 2 Methods

### 2.1 Inclusion and Exclusion Criteria

#### 2.1.1 Type of Study

All randomized controlled trials (RCTs) and observational studies of the clinical use of IVM in the treatment of COVID-19 patients are reported. Studies were not restricted by the year of publication, study site, drug dose, or control group.

#### 2.1.2 Participants

RT-PCR (reverse transcription–polymerase chain reaction) confirmed COVID-19 patients aged over 5 years of both genders were collected and without any serious complications in the study.

#### 2.1.3 Interventions

All interventions included in the study were the use of IVM with COVID-19 patients with the concurrent standard of care or treatment regimens, regardless of the dose, duration, and frequency.

#### 2.1.4 Outcome Indicators

The primary outcome indicators were mortality, length of hospital stay, and negative RT-PCR test response, and the secondary outcome indicators were reduction in viral load or clinical improvement of COVID-19.

#### 2.1.5 Exclusion Criteria

Repeated published literature: literature for which data are lacking or full text is not available and additional exclusion criteria are shown in the flow diagram of the studies retrieved for the review (Figure).

#### 2.1.6 Retrieval Strategy

Comprehensive searches of English databases, including PubMed, EBSCOhost, and Web of Science, and the time of index was from December 2019 to March 2022 for each database. The clinical trial, SR, review, and meta-analysis were all considered for inclusion. Full-text searches were performed using “Ivermectin”, “COVID-19”, or “Ivermectin and COVID-19” as keywords.

#### 2.1.7 Literature Screening

After excluding duplicates, the literature was screened and cross-checked by reading the title, abstract, and full text, according to the inclusion and exclusion criteria. Extracts included the following: first author, country of publication, year of initiation, type of study, intervention/control, study duration, and main findings.

## 3 Results

**FIGURE 1:**
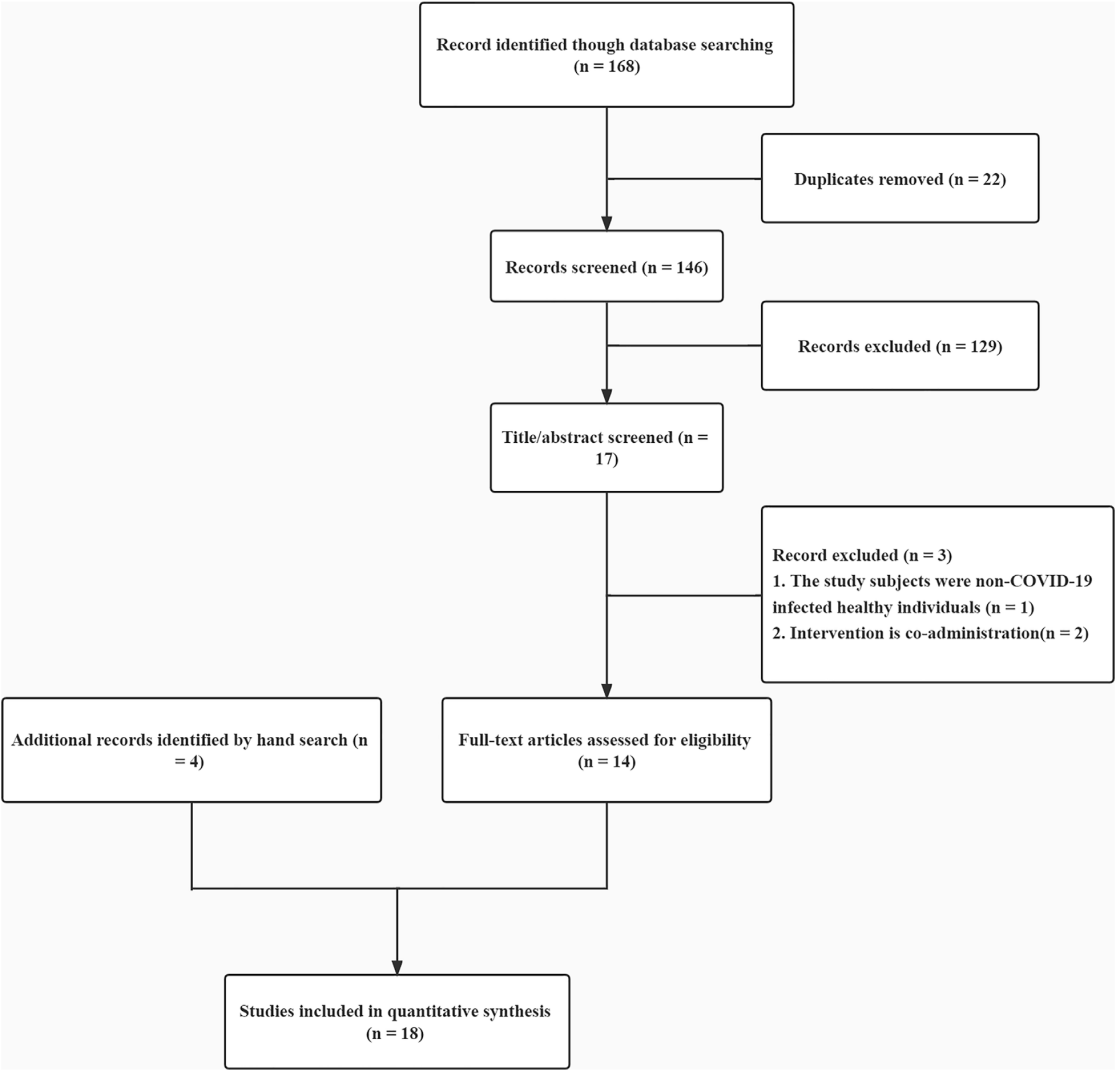
Study flow diagram of literature search.

A total of 168 publications were screened for this review, and a total of 14 studies met the inclusion criteria, with 4 literature studies manually added. A total of 18 studies met the inclusion criteria (*n* = 3,248), of which 15 were randomized controlled trials and three were observational studies with control groups (Figure 1). Included studies were conducted in multiple countries and among different age groups and they varied in size. Eight studies (44%) were conducted in Asia, 2 (11%) in Europe, 3 (17%) in Africa, 2 (11%) in the United States, and 3 (17%) in South America. Among them, 17 studies (94%) had adult patients (age ≥18 years) as participants, and one study from Iran study (6%) required subjects to be > 5 years of age. The duration of 16 of these studies ranged from 5 days to 12 months. Dosing regimens were recorded in the studies, and one study used a nano-suspension nasal spray. In 12 studies, there were no significant differences in demographic and clinical characteristics at baseline. In the other three studies, there were no significant differences or no differences between study groups in blood biochemical characteristics, vital signs, endocrine markers, or complete blood counts. Three other studies did not mention baseline.

### 3.1 Main Outcome

Eleven studies described the main indicators, including mortality, length of hospitalization, and RT-PCR detection of negative reactions. See Table 1 for the basic characteristics of the literature and Table 2 for the relevant data.

**TABLE 1:** Summary of studies of COVID-19 patients on IVM in terms of primary outcomes.

Source	Country (year of launch)	Research sample	Type of study	Comparison	Duration of the study	Main conclusions
Rajter et al. (2021)	United States, 2020	Consecutive hospitalized patients (aged ≥ 18 years) with severe pulmonary involvement diagnosed with SARS-CoV-2 infection (*n* = 280)	Retrospective study	IVM (200 mg/kg) treatment (*n* = 173) vs. standard care (*n* = 107)	58 days	Mortality was significantly lower in the group treated with IVM than in the usual care group
Ravikirti et al. (2021)	India, 2020	All adult patients (aged ≥ 18 years) admitted with a diagnosis of mild to moderate COVID-19 at the hospital (*n* = 112)	Double-blind, randomized controlled trial	Received IVM 12 mg (*n* = 55) vs. placebo tablets (*n* = 57)	8 months–10 months	The IVM group was statistically different from the control group in terms of mortality
Okumuş et al. (2021)	Turkey, 2020	Adult patients with severe COVID-19 pneumonia (*n* = 66)	Randomized controlled trial	The study group (*n* = 36) added IVM 200 mcg/kg/day in addition to the reference treatment vs. the control group (*n* = 30) only received the reference treatment	5 days	No significant difference in mortality between IVM and control groups
Khan et al. (2020)	Bangladesh, 2020	Adult COVID-19 patients without any other serious pathological conditions (*n* = 248)	Retrospective study	IVM 12 mg with standard care (*n* = 115) vs. people receiving SC only (*n* = 133)	5 days	Mortality was significantly lower in the IVM group than in the SC group
Ozer et al. (2022)	United States, 2020	Adult patients who tested positive for SARS-CoV-2 PCR and were diagnosed with COVID-19 pneumonia (*n* = 286)	Prospective study	Patients on standard treatment + two doses of 200 μg/kg IVM (*n* = 60) vs. patients on standard treatment (*n* = 226)	123 days	There was no difference in the length of stay, ICU admissions, intubation rates, and in-hospital mortality between the IVM and control groups
Abd-Elsalam et al. (2021)	Egypt, 2020	All adult patients from ages 20 to 65 with mildly to moderately affected COVID-19 infection confirmed by PCR (*n* = 164)	Randomized controlled trial	Received IVM (12 mg/day) with standard care (*n* = 82) vs. standard care only (*n* = 82)	30 days	There was no statistically significant difference in COVID-19 patients on IVM (12 mg/day for 3 days) at any endpoint
Vallejos et al. (2020)	Argentina, 2020	Patients aged 18 years or older with a diagnosis of COVID-19 confirmed by RT-PCR and weighing equal to or greater than 48 kg (*n* = 501)	Randomized controlled trial	Patients of different weights were divided into three groups and given different doses of IVM with standard treatment group (*n* = 250) vs. the placebo and standard treatment group (*n* = 251)	188 days	Staggered 2-day doses of IVM by patient weight had no significant effect on preventing hospitalization in patients with COVID-19
Mohan et al. (2021)	India, 2020	Consecutive patients older than 18 years with positive results for SARS-CoV-2 RT-PCR or rapid antigen testing for non-severe COVID-19 (*n* = 125)	Double-blind, randomized controlled trial	Single oral formulation of IVM at a dose of 24 mg or 12 mg, or placebo, in a 1:1:1 ratio	A minimum of 14 days or until hospital discharge	A single oral dose of IVM did not significantly increase negative RT-PCR responses
Chaccour et al. (2021)	Spain, 2020	Adult patients with symptoms consistent with COVID-19, with fever or cough not exceeding 72 h, and positive for SARS-CoV-2 by polymerase chain reaction (*n* = 24)	Randomized controlled trial	Single oral dose of IVM (400 μg/kg) (*n* = 12) vs. placebo (*n* = 12)	42 days	There was no difference in the proportion of PCR-positive patients in the IVM and control groups
Ahmed et al. (2021)	Bangladesh, 2020	Inclusion criteria were age 18–65 years; admitted to hospital within the last 7 days; presence of fever (37.5 ≥ C°), cough, and/or sore throat; and diagnosed positive for SARS-CoV-2 by rRT-PCR (*n* = 68)	Randomized controlled trial	Oral IVM alone (12 mg once daily for 5 days) (*n* = 22), oral IVM in combination with doxycycline (12 mg IVM single dose and 200 mg doxycycline on day 1, followed by 100 mg every 12 h for the next 4 days) (*n* = 23), and a placebo control group (*n* = 23)	14 days	5-day course of IVM without co-morbidities showed a faster clearance of SARS-CoV-2 virus than placebo group
Babalola et al. (2022)	Nigeria, 2020	The patient inclusion criteria were COVID 19 PCR proven positive patients and were either asymptomatic or had mild/moderate symptoms (*n* = 62)	Randomized controlled trial	A: IV 6 mg regime (*n* = 21); B: IV 12 mg regime (given Q84 h for 2 weeks) (*n* = 21); and C control: lopinavir/ritonavir (*n* = 20). All groups and the standard of care	14 days	IVM exhibited a dose-dependent significant inhibitory effect on SARS-CoV-2

**TABLE 2:** Summary of statistical analysis data on primary outcomes.

Source	Mortality			Length of hospitalization (day)			RT-PCR detection of negative reactions		
	Intervention group	Control group	*p*-value	Intervention group	Control group	*p*-value	Intervention group	Control group	*p*-value
Rajter et al. (2021)	26/173 (15%)	27/107 (25.2%)	*p* = 0.03	—	*—*	*—*	*—*	*—*	*—*
Ravikirti et al. (2021)	0/55 (0%)	4/57 (7%)	*—*	10 (20.0%)	10 (26.3%)	*p* = 0.429	13/55 (23.6%)	18/57 (31.6%)	*p* = 0.348
Okumuş et al. (2021)	6/36 (20%)	9/30 (30%)	*p* = 0.37	*—*	*—*	*—*	14/16 (87.5%)	3/8 (37.5%)	*p* = 0.01
Khan et al. (2020)	1/115 (0.9%)	9/133 (6.8%)	*p* < 0.05	9 (7–10)	15 (12–19)	*p* < 0.001	4 (4–6) (days)	15 (12–17) (days)	*p* < 0.001
Ozer et al. (2022)	*—*	*—*	*—*	7	6	*p* = 0.06	*—*	*—*	*—*
Abd-Elsalam et al. (2021)	3/82 (3.7%)	4/82 (4.9%)	*p* = 1.00	8.82 ± 4.94	10.97 ± 5.28	*p* = 0.085	*—*	*—*	*—*
Vallejos et al. (2020)	4/250 (1.60%)	3/251 (1.20%)	*p* = 0.72	*—*	*—*	*—*	212/250 (89.08%)	221/251 (92.47%)	*p* = 0.29
Mohan et al. (2021)	*—*	*—*	*—*	*—*	*—*	*—*	14/40 (35.0%)	14/45 (31.1%)	*p* = 0.82
Chaccour et al. (2021)	*—*	*—*	*—*	*—*	*—*	*—*	1/12 (91%)	0/12 (0%)	*—*
Ahmed et al. (2021)	*—*	*—*	*—*	9.6	9.7	*—*	9.7 (days)	12.7 (days)	*p* = 0.02
Babalola et al. (2022)	*—*	*—*	*—*	*—*	*—*	*—*	4.65 (days)	9.15 (days)	*—*
López-Medina et al. (2021)	0/200 (0%)	1/200 (0.5%)	*—*	*—*	*—*	*—*	*—*	*—*	*—*
Aref et al. (2021)	*—*	*—*	*—*	*—*	*—*	*—*	54/57 (94.7%)	43/57 (75.4%)	*p* = 0.004
Samaha et al. (2021)	*—*	*—*	*—*	10 (0%)	10 (6%)	*—*	*—*	*—*	*—*
Shahbaznejad et al. (2021)	*—*	*—*	*—*	7.1	8.4	*p* = 0.016	6/17 (35.3%)	3/8 (35.7%)	*—*
Lim et al. (2022)	3/241 (1.2%)	10/249 (4.0%)	*p* = 0.09	7.7	7.3	*p* = 0.38	*—*	*—*	*—*

#### 3.1.1 Mortality

IVM was associated with reduced mortality in three retrospective studies and two RCTs. See Table 1 for details. In the retrospective studies from Florida and Bangladesh (*n* = 528) (Rajter et al., 2021; Khan et al., 2020), patients in the study group were treated with IVM in addition to standard care (SC), whereas patients in the control group were only provided with SC. The final results showed that the mortality treated with IVM was significantly lower than that of patients treated with SC only. The study from eastern India (*n* = 112) (Ravikirti et al., 2021) compared the IVM group with a placebo group and found a statistically significant difference in mortality (*p* = 0.045). IVM was administered at a dose of 200 μg/kg/day in the IVM group in a Turkish study (*n* = 66) (Okumuş et al., 2021), and the control group received only reference therapy without IVM. The investigators in the trial concluded by comparing data from the two study groups that IVM used in patients with severe COVID-19 could increase clinical recovery rates and improve prognostic laboratory parameters; it can reduce mortality, but it is not statistically significant (*p* = 0.37). In the prospective study conducted in the United States (*n* = 286) (Ozer et al., 2022), the IVM group received standard treatment with two additional doses of IVM 200 μg/kg compared to the control group, and after 10 days, the observation of key outcome indicators revealed no difference between the two groups in terms of length of stay and in-hospital mortality.

#### 3.1.2 Length of Hospitalization

See Table 1 for specific research. Two RCTs from Egypt and Argentina (*n* = 665) (Abd-Elsalam et al., 2021; Vallejos et al., 2020) showed no significant difference in the length of hospitalization between the IVM group (which received a certain dose of IVM) and the control group.

#### 3.1.3 RT-PCR Detection of Negative Reactions

The two RCTs included in the review both found no correlation between IVM and negative responses to RT-PCR testing. See Table 1 for more details. In the randomized controlled study in India (*n* = 125) (Mohan et al., 2021), patients hospitalized with mild to moderate COVID-19 were divided into three groups for a single oral formulation of IVM at a dose of 24 mg or 12 mg, or placebo in a 1:1:1 ratio, and it was ultimately found that the IVM group did not significantly increase negative RT-PCR responses. In a randomized controlled study conducted in Spain (*n* = 24) (Chaccour et al., 2021), patients received a single oral dose of IVM (400 μg/kg) and placebo, followed by RT-PCR, which revealed no difference in the proportion of PCR-positive patients between the IVM and control groups.

Researchers in a study from Bangladesh (*n* = 68) (Ahmed et al., 2021) divided the COVID-19 patients included in the study into an oral IVM alone group, oral IVM combined with the doxycycline group, and a placebo control group. The results showed that in the absence of co-morbidity, a 5-day course of IVM treatment showed faster SARS-CoV-2 virus clearance than the placebo arm. In a recent study from Nigeria, to explore the efficacy and safety of IVM in patients with neocoronavirus infection (*n* = 62) (Babalola et al., 2022), the results showed that intravenous administration significantly reduced the number of days of neocoronavirus negative (DTN) in a dose-dependent manner. The investigators concluded that twice-weekly IV 12 mg may be more effective than twice-weekly IV 6 mg and certainly more effective than the non-IV group in the study and that IV should be considered for clinical management of SARS-CoV-2 (Table 1).

For primary outcomes, mortality studies include the following: seven studies showed lower mortality in the IVM group than in the control group (87.5%), with two results statistically different (*p* < 0.05). The duration of patient hospitalization is as follows: six IVM groups had a significantly lower number of hospitalizations within 10 days or length of hospitalization than the control group (75%), with two of them being statistically significantly different (*p* < 0.001 and *p* = 0.016). RT-PCR test: the results of eight studies showed that the IVM group had more negative PCRs than the control group within the study time or took significantly less time to change from positive to negative than the control group (80%), with three of the results being statistically significantly different (*p* < 0.01 and or *p* = 0.01).

### 3.2 Secondary Outcome

Seven studies described the secondary indicators, including a reduction in viral load or clinical improvement of COVID-19. See Table 3 for more details. Among them, a randomized controlled study (*n* = 400) conducted in Colombia (López-Medina et al., 2021) was conducted using IVM at 300 μg/kg body weight per day versus placebo control, and treatment with IVM obtained at the end of the final follow-up did not reduce the course of COVID-19 patients. In a study conducted in Malaysia (*n* = 490) (Lim et al., 2022), the investigators used a daily dose of IVM of 0.4 mg/kg body weight for the IVM group, which was controlled against the standard care to observe the progression of severe disease, and concluded that the IVM treatment during early disease did not prevent the progression. Researchers in Italy administered two different high IVM doses to participants (*n* = 93) with initial, asymptomatic, or oligosymptomatic SARS-CoV-2 infection (Buonfrate et al., 2022) and determined whether taking IVM at a safe dose reduced the viral load of SARS-CoV-2 at day 7. The results showed that the high dose of IVM was safe but did not show a reduction in viral load. In contrast, four studies conducted in Egypt, Lebanon, Iran, and Argentina (*n* = 329) (Aref et al., 2021; Samaha et al., 2021; Shahbaznejad et al., 2021; Krolewiecki et al., 2021) controlled by IVM treatment with other standard treatments and the results of the studies found that IVM rapidly cleared the virus, reduced viral load, and improved severe clinical symptoms in patients with COVID-19.

**TABLE 3:** Summary of studies in COVID-19 patients with IVM in terms of secondary outcomes.

Source	Country (year of launch)	Research sample	Type of study	Comparison	Duration of the study	Main conclusions
López-Medina et al. (2021)	Colombia, 2020	Adult patients with mild illness and no more than 7 days of symptoms (at home or in hospital) (*n* = 400)	Double-blind, randomized controlled trial	Patients received IVM at 300 μg/kg body weight per day for 5 days (*n* = 200) vs. placebo (*n* = 200)	7 months–12 months	The results of the study suggested that IVM does not significantly affect the course of early COVID-19
Aref et al. (2021)	Egypt, 2021	Adult patients diagnosed with mild COVID-19 (*n* = 114)	Randomized controlled trial	Patients received IVM nanosuspension nasal spray with Egyptian COVID-19 regimen (*n* = 57) vs. Egyptian COVID-19 regimen only (*n* = 57)	Follow-up until all COVID-19 patients have fully recovered	Topical treatment of mild COVID-19 patients with IVM nanosuspension nasal spray resulted in rapid viral clearance and reduced asymptomatic duration
Samaha et al. (2021)	Lebanon, 2020	Asymptomatic Lebanese adult patients who tested positive for SARS-CoV-2 (*n* = 100)	Randomized controlled trial	The experimental group (*n* = 50) was treated with standard prophylaxis with a single dose of IVM treatment vs. the control group (*n* = 50) who received standard prophylaxis	10 days	IVM reduces the incidence of severe symptoms and significantly lowers the viral load
Shahbaznejad et al. (2021)	Iran, 2020	Patients with moderate to severe COVID-19 (age > 5 years; weight > 15 kg) (*n* = 70)	Randomized controlled trial	The intervention group received a single weight-based dose (0.2 mg/kg) of IVM (*n* = 35) vs. the control group received the standard of care (*n* = 35)	69 days	The difference between the IVM and control groups was statistically significant (*p* = 0.007). A single body weight dose (0.2 mg/kg) of IVM improved important clinical symptoms in patients with neocoronary pneumonia
Krolewiecki et al. (2021)	Argentina, 2020	COVID-19 patients aged 18 to 69 years old with RT-PCR-confirmed infection, hospitalized and not requiring intensive care (*n* = 45)	Randomized controlled trial	Patients in the IVM group received 5 consecutive days of oral treatment with 600 μg/kg/day IVM (*n* = 30) vs. the untreated control group (*n* = 15)	115 days	Mean IVM plasma concentration levels were positively correlated with the viral decay rate
Lim et al. (2022)	Malaysia, 2021	The patients 50 years and older with laboratory-confirmed COVID-19, comorbidities, and mild to moderate disease (*n* = 490)	Randomized controlled trial	Patients were randomized in a 1:1 ratio to receive either oral IVM, 0.4 mg/kg body weight daily for 5 days, with the standard of care (*n* = 241) vs. the standard of care alone (*n* = 249)	28 days	IVM treatment during early illness did not prevent progression to severe disease
Buonfrate et al. (2022)	Italy, 2021	Participants were adults recently diagnosed with asymptomatic/oligosymptomatic SARS-CoV-2 infection (*n* = 93)	Randomized controlled trial	placebo (arm A) (*n* = 32); single dose IVM 600 μg/kg and placebo for 5 days (arm B) (*n* = 29); and single-dose IVM 1200 μg/kg for 5 days (arm C) (*n* = 32)	30 days	High-dose IVM was safe but did not show efficacy to reduce viral load

### 3.3 Adverse Reactions

During the trial conducted in Argentina (Vallejos et al., 2020), there were 45 non-serious adverse events such as pneumonia, diarrhea, and fever in the IVM group, and no serious adverse events occurred. In the Spanish study (Chaccour et al., 2021), patients in the IVM group experienced adverse reactions with dizziness and blurred vision as the main symptoms. In a trial conducted in Argentina (Krolewiecki et al., 2021), the most common adverse events found during the study in the IVM group were three cases of mild rash; the control group was only a single event of abdominal pain, dizziness, anxiety, pain, and hyperglycemia (all mild). The most common adverse event in the Malaysian study (Lim et al., 2022) was diarrhea [14 cases (5.8%) in the IVM group and four cases (1.6%) in the control group]. There were 229 AEs reported in the study conducted in Italy (Buonfrate et al., 2022), with the most frequent adverse events involving transient eye disease, followed by neurological disorders, fatigue, and gastrointestinal symptoms. Four AEs were recorded as serious (SAEs): in all cases, they required hospitalization for worsening of the disease with no causal relationship to the study drug. All events resolved. No adverse events have been reported in other trials.

## 4 Discussion

The oral formulation of IVM is currently used in clinical practice; to allow only a high concentration of the drug at the site of action, some experts have proposed considering IVM inhalation therapy as an approach. A prospective study was conducted by Zaki F Aref (López-Medina et al., 2021) in Egypt in patients with mild COVID-19 treated with topical IVM nanosuspension nasal spray and showed rapid viral clearance and anosmia duration. Some experts have proposed changing inhalation administration; data from animal trial studies, such as safety and tolerability, need to be refined before the new inhaler can be exposed to humans and whether it has the ideal inhalation properties and the possible effects on drug concentrations needs to be evaluated (Schmith et al., 2020), and the aforementioned analysis can provide a reference for future new drug development. Patients are not recommended to take IVM without knowing the drug dose to avoid adverse events.

No serious adverse events were reported in all the literature reports, but there were reactions such as dizziness, blurred vision, and rash. In a report from the Oregon Poison Center, 21 patients developed toxicity in August due to using IVM for the prevention or treatment of COVID-19; these cases illustrated the potential toxic effect of IVM, including severe episodes of confusion, ataxia, seizures, and hypotension (Temple et al., 2021). IVM is more widely used in Latin America, and some studies have observed common toxic reactions such as diarrhea, dizziness, abdominal pain, and vomiting. Physicians have reported an increasing number of cases of IVM-associated hepatitis (Molento 2021). A survey shows that by the beginning of 2021, dispensing rates regarding IVM have increased in all regions of the United States, but there are insufficient data to support or oppose the recommendation for IVM treatment of COVID-19 (Lind et al., 2021). Physicians need to remain cautious when administering IVM to COVID-19 patients in actual clinical practice, determining the most effective IVM dose, combination, and timing for their patients so as not to compromise patient outcomes. It is necessary to determine the most effective dose, combination, and timing of IVM for the patient as it may affect the patient’s outcome to some extent (Wehbe et al., 2021).

Of the 18 clinical trials collected in this study, results showed that the use of IVM is slightly better than other regimens in terms of mortality, length of hospitalization, and RT-PCR conversion rate. These studies are not powered sufficiently to detect differences in the secondary outcomes, so a positive conclusion could not be reached.

### 4.1 Limitations

This review has several limitations. First, the effectiveness of IVM is controversial due to the lack of science-based treatment guidelines, and it is not recommended as a treatment or prophylactic agent for COVID-19 in many countries. Only well-designed and reported study analyses can provide valuable confirmatory information for clinical administration (Roman et al., 2022). Second, the number of studies collected for this review is small, so there could be different ideas about the conclusion, and more RCTs will be needed for analysis. Third, the data from studies may be biased as they covered a limited range of countries and the patient’s ages. Types of COVID-19 pneumonia and initial vital signs were not fully informed.

## 5 Conclusion

In summary, IVM may be effective for treating mildly to moderately ill patients, but the result is still in the early stages of clinical application as an antiviral drug and whether it has definite efficacy against COVID-19 needs to be supported by more controlled clinical studies with large samples. Therefore, self-medication is not recommended for COVID-19 patients. Clinicians must take IVM with caution based on high-level evidence and benefit-risk assessment results.

## Data Availability

The original contributions presented in the study are included in the article/supplementary material; further inquiries can be directed to the corresponding author.
